# Sensitive and Rapid Detection of Aspartic Acid with Co_3_O_4_-ZnO Nanorods Using Differential Pulse Voltammetry

**DOI:** 10.3390/bios13010088

**Published:** 2023-01-05

**Authors:** Sulaiman Y. Alfaifi, Waheed Abiodun Adeosun, Abdullah M. Asiri, Mohammed M. Rahman

**Affiliations:** 1 Chemistry Department, Faculty of Science, King Abdulaziz University, P.O. Box 80203, Jeddah 21589, Saudi Arabia; 2 Center of Excellence for Advanced Materials Research (CEAMR), King Abdulaziz University, P.O. Box 80203, Jeddah 21589, Saudi Arabia

**Keywords:** Co_3_O_4_-ZnO nanorods, aspartic acid, electrochemical method, sensitivity, healthcare safety

## Abstract

Herein, the detection of aspartic acid by doped Co_3_O_4_-ZnO nanorod materials was proposed using differential pulse voltammetry. The nano-composite metal oxide was synthesized by the wet precipitation method in basic media. Aspartic acid is a non-essential amino acid naturally synthesized in the body with lot of health significance, including as a biomarker for several health deficiencies. The synthesized composite Co_3_O_4_-ZnO nanorod was well-investigated by using FESEM, XRD, XPS, FTIR, UV/vis., EIS, and CV. The synthesized composite exhibited a low limit of detection (0.03 µM, high sensitivity (0.0014 µA µM^−1^ cm^−2^) and wide linear range (0.05–50 µM) for aspartic acid. The substrate, the Co_3_O_4_-ZnO nanorod, enhanced the electro-catalytic oxidation of aspartic acid as a result of its catalytic and conductivity properties. The developed sensor based on Co_3_O_4_-ZnO has a repeatable, reproducible and stable current response for aspartic acid. Additionally, other electroactive compounds did not interfere with the sensor’s current response. The suitability of the developed sensor for real sample analysis was also established. Therefore, this study proposed the potential use of Co_3_O_4_-ZnO nanorod material in healthcare management for the maintenance of human well-being.

## 1. Introduction

Amino acids are an important organic biochemical in human metabolism. They are among the vital nutrients necessary for body building and general well-being [1]. An important class of amino acid is aspartic acid, which is naturally synthesized in the body. It is also synthesized in the body though oxaloacetate [2]. Another notable source of aspartic acid is sugar cane. Aspartic acid helps in muscle strengthening and enduring strength through the generation of luteinizing and testosterone hormones. In addition, aspartic acid is an important neurological transmitter in the central nervous system. The concentration of aspartic acid in the body could be a biomarker for lung and neck cancer, Alzheimer’s syndrome, Lenox syndrome and epilepsy [2,3]. Due to its physiological and biological importance, several techniques have been proposed for measuring aspartic acid in biological samples. Such methods include chromatography (ion exchange, high-performance liquid chromatography and gas chromatography), fluorescence, spectrophotometry and electrochemical routes (enzyme and non-enzyme-based) [4,5,6,7]. Methods based on chromatography and spectrophotometry are very sensitive but suffer major setbacks because of their long analysis time, tedious sample preparation and high cost of analysis. In contrast, electrochemical methods offer a low cost of analysis, high accuracy and simplicity, which makes the electrochemical method highly attractive [8,9,10]. Gao et al., in their studies, developed an aspartic acid detector using a Cu (II)-catalyzed oscillating reaction. This method is based on the reaction between H_2_O_2_ and sodium thiocyanate catalyzed by Cu(II) ions. There was a low limit of detection (5.58 × 10^−8^ mol L^−1^) in the linear range of 1.17 × 10^−5^ to 7.0 × 10^−8^ mol L^−1^ [11].

In addition, Fontanarosa and his research group reported the very sensitive detection of aspartic acid with a tandem mass spectrometer. Their study reported a very low limit of detection (0.52 pg/µL) [12]. Similarly, Tabaraki et al. developed a fluorescence-based method for the assay of aspartic acid in human serum. They employed n-doped carbon dots synthesized with the aid of microwave radiation as a fluorescent. The quenching of the fluorescence upon the addition of aspartic acid forms the basis of the sensing. Their method reported a low limit of detection (90.0 nM) in the linear range of 0.5–50 µM [1].

Prasad and his team worked on the fabrication of an electrochemical sensor for D- and L- aspartic acid using molecularly imprinted poly (indole 3-acetic acid)/multiwalled carbon nanotube/pencil graphite electrode. The determination of D-aspartic acid and L-aspartic acid was conducted by differential pulse anodic stripping voltammetry. The detection limits of 0.025 µM and 0.016 µM were reported for D- and L- aspartic acid, respectively [3]. The aforementioned methods offer good performance for aspartic acid detection, but the need for a cheap, sensitive procedure suitable for in situ analysis necessitates further research. This study, therefore, explores the electrochemical method for the detection of aspartic acid in real samples using a metal oxide nanomaterial composite. Co_3_O_4_ is a metallic oxide and a semi-conductor which is very interesting due to its electronic, magnetic, optical and electrochemical properties [13,14,15,16,17]. In addition, Co_3_O_4_ has good catalytic properties which makes it an ideal material for electrochemical sensing. Likewise, ZnO is another metal oxide and n-type semi-conductor with unique material properties. It has a direct band-gap of 3.5 eV and wide excitation binding energy (60.0 meV), as well as good catalytic properties. ZnO has interesting applications in electro-catalysis, energy storage and electrochemical sensing [17,18,19,20]. The composition of these two materials has been reported to have excellent applications in electrocatalysis. While there are a plethora of studies on the individual synthesis of ZnO and Co_3_O_4_, very few studies report the synthesis of Co_3_O_4_-ZnO nanorods. For instance, Tak et al. reported the synthesis of Co_3_O_4_-ZnO nanorods by the photochemical coating method. In their studies, ZnO nanowires were first synthesized by the hydrothermal method and Co_3_O_4_ was then coated on ZnO nanowires by the photochemical reaction [21].

Moreover, Reda and his research group reported Co_3_O_4_-ZnO nanorod synthesis by the facile solid-state grinding technique [22]. The synthesized hetero-structure nanocomposite was studied for its photocatalytic property. Other studies by Yang et al. [20], Liu et al. [23], Sharma et al. [16], and Xu et al. [24] have reported successful synthesis of Co_3_O_4_-ZnO low-dimensional nanorods. Based on the simplicity and generation of highly structured material, the simple wet chemical method was employed for the synthesis of the Co_3_O_4_-ZnO nanorods used for this study. This study therefore reports for the first time the electrochemical detection of aspartic acid using Co_3_O_4_-ZnO nanorods, which makes this study an important and a novel study. Additionally, the prospect or suitability of the proposed sensor for real sample analysis was measured and established.

## 2. Materials and Methods

### 2.1. Chemicals and Reagents

The essential chemicals used for the proposed experiment include cobalt II nitrate (Sigma Aldrich, New York, NY, USA), zinc II sulfate (Sigma Aldrich, New York, NY, USA), ammonia solution (25%) (Sigma Aldrich, New York, NY, USA), aspartic acid (Sigma Aldrich, New York, NY, USA), sodium dihydrogen phosphate, disodium hydrogen phosphate, potassium chloride, glucose, nitrite, melamine, glutamic acid, leucine, uric acid and ascorbic acid. All of the chemicals used for this study were of analytical grade and were used as bought without any further treatment.

### 2.2. Apparatus

The apparatus used for this study includes a field emission scanning electron microscope (FESEM, JSM-7600F, JEOL, Tokyo, Japan) fitted with an X-ray electron diffraction spectrometer (Thermo Scientific, Tokyo, Japan), X-ray photoelectron spectrometer (XPS) (Thermo Scientific, Tokyo, Japan), Fourier transform infrared spectrometer (FTIR) (X-Vision, Tokyo, Japan), ultraviolet–visible spectrophotometer (UV-Vis.) (Thermo Scientific, Tokyo, Japan) and potentiostat powered by Autolab Nova 2.1 (Tokyo, Japan) comprising working, reference and counter electrodes. The working electrode at any point in time was a glassy carbon electrode or bare electrode, while the reference and counter electrodes were made up of Ag/AgCl (3 M KCl) and platinum wire, respectively.

### 2.3. Synthesis of Co_3_O_4_-ZnO Nanorods

The facile wet chemical method was used for the synthesis of Co_3_O_4_ as described. A total of 0.1 M Co(NO_3_)_3_·6H_2_O (2.91 g/100.0 mL) was prepared. Then, 80.0 mL of the solution was measured into a 250.0 mL beaker. Additionally, 80.0 mL 0.1 M ZnSO_4_·2H_2_O (1.97 g/100.0 mL) was prepared and added to the beaker containing 80.0 mL Co_3_O_4_. The two mixtures were then stirred for about 90 min upon the dropwise addition of 20.0 mL ammonia solution (25%). After 90 min of continuous stirring at 80 rpm, maintaining a temperature of 80 °C, a pinkish precipitate was formed and well-washed with deionized water followed by acetone. The washed precipitate was completely dried in the oven overnight at 80 °C. The dried powdery material obtained was then properly ground using a mortar and pestle. Lastly, the well-ground powdery sample was calcined in the muffle furnace at 600 °C for 3 h. After calcination, the color of the prepared sample changed from pink to black.

### 2.4. Fabrication of Co_3_O_4_-ZnO Nanorod Coated Electrode

The glassy carbon electrode was first soaked in ethanolic solution for about an hour and was then cleaned by rubbing on a micro-cloth pad soaked with 0.5 µm alumina slurry. It was then washed and rinsed with de-ionized water [25]. In addition, chemically adsorbed material on the GCE surface was removed using cyclic voltammetry (0.25 M sulfuric acid as supporting electrolyte). About 30.0 µg of Co_3_O_4_-ZnO nanorod was then measured into a watch glass with the addition of 10 µL of ethanolic solution to disperse the nanoparticle. The dispersed nanoparticle was then applied directly on the GCE surface and was cemented using one drop of nafion solution (conducting binder). The prepared Co_3_O_4_-ZnO nanorod was then allowed to dry in the open air for about 2 h and used for further studies.

### 2.5. Characterization Technique

The surface morphology of the synthesized metallic oxide nanocomposite was assessed using FESEM (tungsten filament, pictures taken using low and high magnifications). The binding energy and elemental composition were obtained using X-ray photoelectron spectroscopy (at the binding energy range of 0–1400 eV). Additionally, XEDS fitted with FESEM was used for analyzing the elemental composition in the synthesized nanocomposite at an electron beam size of 10.0 µm. Optical studies were conducted to determine the band gap and elemental functionality (functional groups) using UV-Vis and FTIR spectrometers. Electrochemical characterization studies such as electrochemical impedance spectroscopy as well as cyclic voltammetry were conducted using a potentiostat. For electrochemical impedance spectroscopy, an amplitude of 5.0 mV and DC potential of +0.2 V were applied. Additionally, cyclic and linear sweep voltametric studies were carried out at a scan rate of 100 mV/s, with a potential window of 0 to 1.0 V, modulation time of 0.0024 s and step potential of 0.008 V.

### 2.6. Interference Studies

Likely interferents such as electroactive molecules, including ascorbic acid, uric acid, glucose, nitrite, etc., were investigated for interference with the oxidation current given by the Co_3_O_4_-ZnO nanorod. Other factors such as repeatability and reproducibility were duly investigated.

### 2.7. Real Sample Analysis

As-purchased mouse serum was used for real sample analysis. The collected samples were diluted by 10-fold using phosphate buffer (0.1 M PBS-pH 5.7) to mask the effect of the matrix. The standard addition method was used for the detection as well as the determination of the spiked sample.

## 3. Results and Discussion

### 3.1. Synthesis of Co_3_O_4_-ZnO Nanorod

Synthesis of the Co_3_O_4_-ZnO nanorod was thought to have occurred through Ostwald ripening. The obtained Co_3_O_4_ was crystalline, which suggests that particle growth is dominated by nucleation during formation. The obtained metal oxide composite was pinkish after drying and turned black upon calcination.

### 3.2. Structural and Morphological Studies

**Figure 1:**
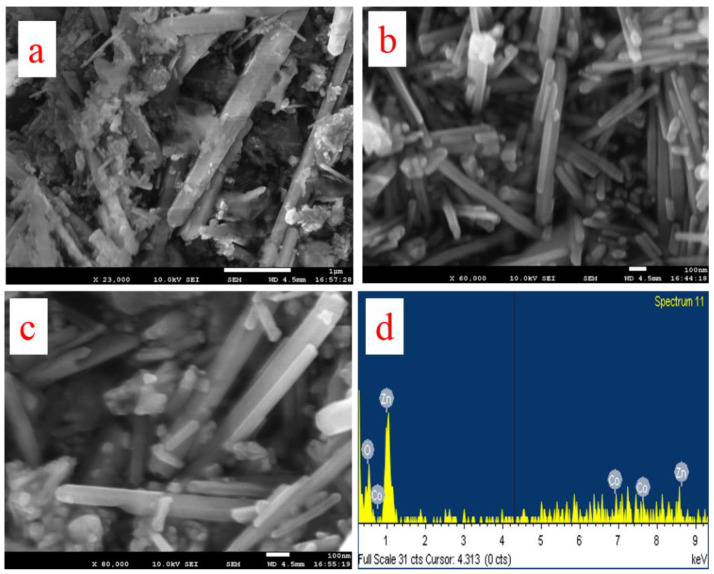
Morphological and elemental analyses. (**a**–**c**) FESEM images of Co_3_O_4_-ZnO. (**d**) XEDS spectrum of Co_3_O_4_-ZnO.

FESEM analysis was conducted to reveal the morphology and microstructure of the synthesized nanocomposite. The obtained images are presented in Figure 1. The structural property of a nanomaterial could influence the material property of the material, such as the surface area, which may eventually have an effect on the performance of the nanomaterial in applications such as electrocatalysis. FESEM images revealed nanorods (app 30–60 nm) wrapped with semi-crystalline whitish material (Figure 1a–c). The nanorods could be associated with Co_3_O_4_ while the whitish patches could be associated with ZnO. ZnO is thought to consist of agglomerated nanoparticle lumps with a denser distribution while Co_3_O_4_ has a nanorod structure with high crystallinity [20].

### 3.3. XEDS Analysis

XEDS is an important characterization tool for the investigation of elemental composition in a material. The obtained XEDS spectrum is shown in Figure 1d. The result indicates the presence of cobalt, zinc and oxygen.

### 3.4. XPS Analysis

**Figure 2:**
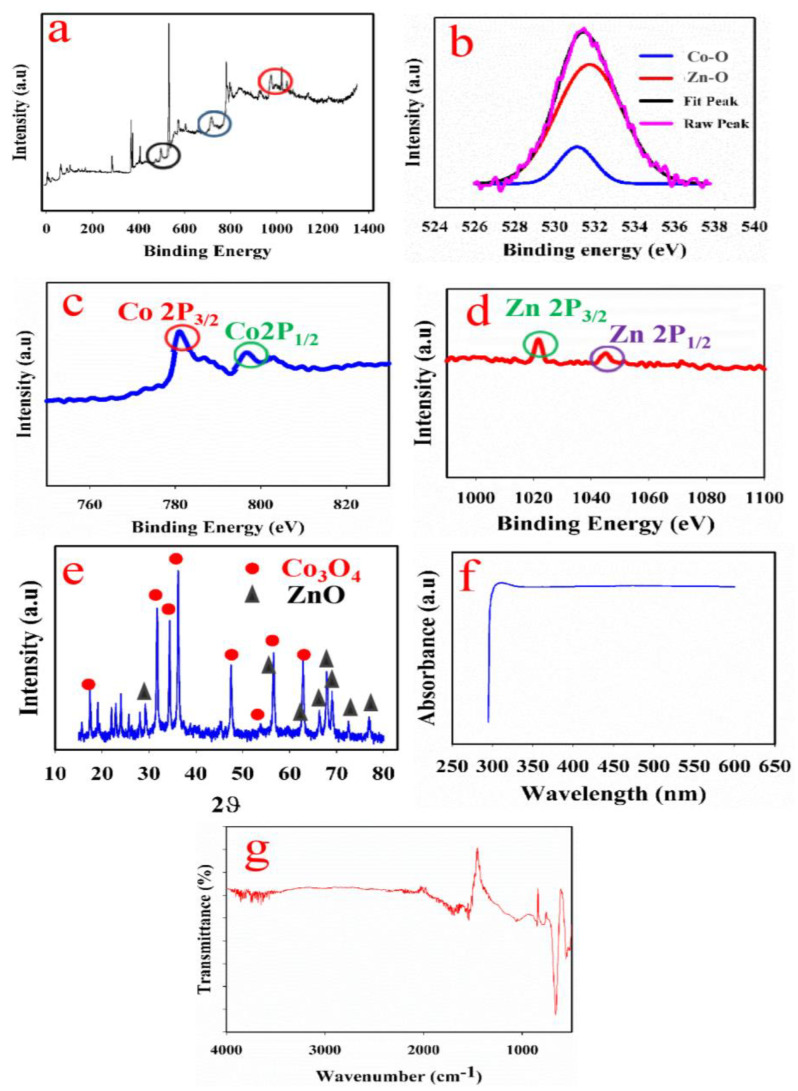
Optical, functional and structural analyses. (**a**) Full XPS spectrum of Co_3_O_4_-ZnO nanorod. (**b**) Deconvoluted peak of oxygen. (**c**) Deconvoluted peak of cobalt. (**d**) Deconvoluted peak of zinc. (**e**) XRD spectrum of Co_3_O_4_-ZnO nanorod. (**f**) Obtained UV-Vis spectrum for Co_3_O_4_-ZnO nanorod. (**g**) FTIR spectrum of Co_3_O_4_-ZnO nanorod.

XPS, also called electron spectroscopy for chemical analysis (ESCA), is a surface analysis technique that is commonly utilized for quantitatively investigating the chemical state of elemental composition in a material. The XPS spectrum obtained is given in Figure 2a. The full spectrum indicates peaks at the regions of 530, 782 and 1030, corresponding to cobalt, zinc and oxygen, respectively. The binding energies at 529.8 eV and 531.4 eV could be attributed to the O 1s spectrum of the Co_3_O_4_-ZnO nanorod (Figure 2b). The peak at 529.9 eV (low energy oxygen peak) could be assigned to lattice oxygen (O^2−^) in the Co_3_O_4_-ZnO, while the peak at 531.4 eV could be linked to chemisorbed oxygen and surface hydrolysis. The XPS result indicates the presence of Co_3_O_4_ and ZnO in the prepared nanocomposite. Deconvoluted peaks at 781.3 and 782.4 eV could be attributable to Co2P_3/2_ and Co 2P_1/2_, respectively (Figure 2c). Additionally, the deconvoluted peaks at 1021.6 eV and 1044.95 eV (Figure 2d) are attributable to the binding energy of Zn 2P_3/2_ and Zn 2P½, respectively [16,20,26].

### 3.5. XRD Analysis

XRD analysis provides information about the chemical composition of materials as it gives information on the crystalline phases of the component materials. The observed XRD spectrum for the Co_3_O_4_-ZnO nanorod is given in Figure 2e. Diffraction peaks at (2θ) 19°, 31.20°, 35.48°, 37.80°, 43.2°, 54.89°, 59.20° and 63.01° corresponding to diffraction lines of (111), (220), (311), (222), (400), (422), (511) and (440), respectively, are typical for the cubic spinal structure of Co_3_O_4_ (JCPDS No. 42.1467). Moreover, diffraction peaks at 2θ degree, 34.50°, 56.75°, 62.50°, 68.01°, 69.05°, 72.61° and 76.50°, correspond to diffraction lines of (002), (110), (103), (112), (201), (004) and (202), respectively (JCODS No.79-2205). The above diffraction patterns established the successful formation of Co_3_O_4_-ZnO nanorods [16,27]. The size of the nanocomposite crystal was evaluated using the Derbye–Sherrer equation, as presented in Equation (1) [16].

δ = 0.89λ/βcos θ(1)

where δ denotes crystallite size, λ denotes the wavelength of Cu-Kα (given as 0.15 nm), β denotes full width at half maximum of the selected peak, and θ denotes the diffraction angle. The crystallite size of the nanocomposite is 42.0 nm.

### 3.6. Optical Analysis (UV-Vis and FTIR Analyses)

UV-Vis spectroscopic analysis was conducted to determine the band structure and energy in Co_3_O_4_-ZnO nanorods. The obtained UV-Vis spectrum is presented in Figure 2f. The spectrum shows bands at 374 nm attributable to the Co_3_O_4_-ZnO nanorod, conforming with previous studies [16].

The obtained FTIR spectrum is given in Figure 2g. The observed peak at 550 cm^−1^ corresponds to Co-O vibration in Co_3_O_4_ [28,29,30]. Usually, characteristic bands in the region of 600–1000 cm^−1^ are associated with vibration of metallic ions in the crystal lattice. Similarly, the peaks noticed in the region of 390 cm^−1^ could be attributed to Zn-O in ZnO [17,31,32]. Therefore, the observed FTIR analysis suggests the presence of Co_3_O_4_ and ZnO in the prepared nanorod.

### 3.7. Electrochemical Characterization

#### 3.7.1. Electron Mobility

**Figure 3:**
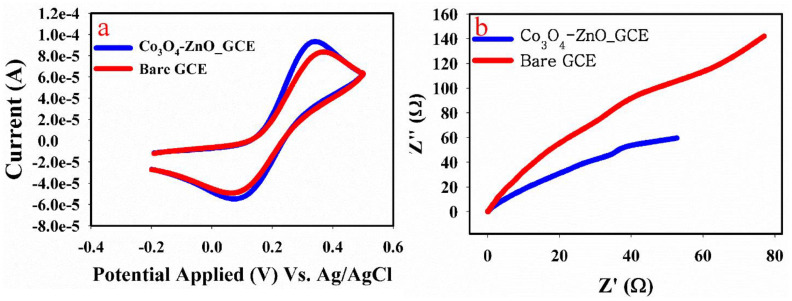
(**a**) Cyclic voltammetric response of Co_3_O_4_−ZnO nanorod modified GCE in 1 mM Fe (CN)_6_^3−/4−^ at the scan rate of 75 mV/s. (**b**) EIS spectrum of Co_3_O_4_−ZnO nanorod in 0.1 M PBS 5.7/0.1 µM aspartic acid.

Electron mobility on the surface of the GCE was accessed by cyclic voltammetry in the ferricyanide couple (Fe(CN)_6_^3−/4−^). The cyclic voltammetry sweep in 1 mM Fe(CN)_6_^3−/4−^ was conducted in the window range of −0.2 to 0.5 V at a scan rate of 75 mV/s. The obtained result is given in Figure 3a. From the result, it could be observed that a higher oxidation current was recorded with the coated GCE (0.1 mA) compared with bare GCE (80 µA). The increased current indicates improved electron mobility on the surface of GCE [33,34]. Likewise, the peak to peak potential (∆E_p_) of the Co_3_O_4_-ZnO nanorod modified GCE was smaller (140 mV) compared with the bare GCE (200 mV). This is also an indication of higher electron mobility on the Co_3_O_4_-ZnO nanorod modified GCE surface.

#### 3.7.2. Impedance Study

Electrochemical impedance spectroscopy (EIS) was conducted to assess the resistivity between the electrolyte and electrode interface. The obtained spectrum is given in Figure 3b. In EIS, the semi-circle denotes charge transfer resistance (Rct), which is associated with electron/charge mobility on the surface of the electrode. The smaller the Rct, the better the electron mobility and vice versa [35,36,37,38]. As shown in Figure 3a, a smaller Rct was obtained for the Co_3_O_4_-ZnO nanorod (219 kΩ) as compared with bare GCE (669 kΩ). This indicates that the substrate on the GCE (Co_3_O_4_-ZnO nanorod) improves electron mobility on the electrode surface. This property is also essential for the electrocatalytic properties of the Co_3_O_4_-ZnO nanorod. The equivalent circuit diagram for the EIS study is presented in Figure 3b and Electronic Supplemental Section S1, where Rs is the solution resistance, CPE denotes the constant phase element, W is the Warburg constant and Rct/Rp is the charge transfer resistance.

### 3.8. Sensing Application

#### 3.8.1. Control Study

**Figure 4:**
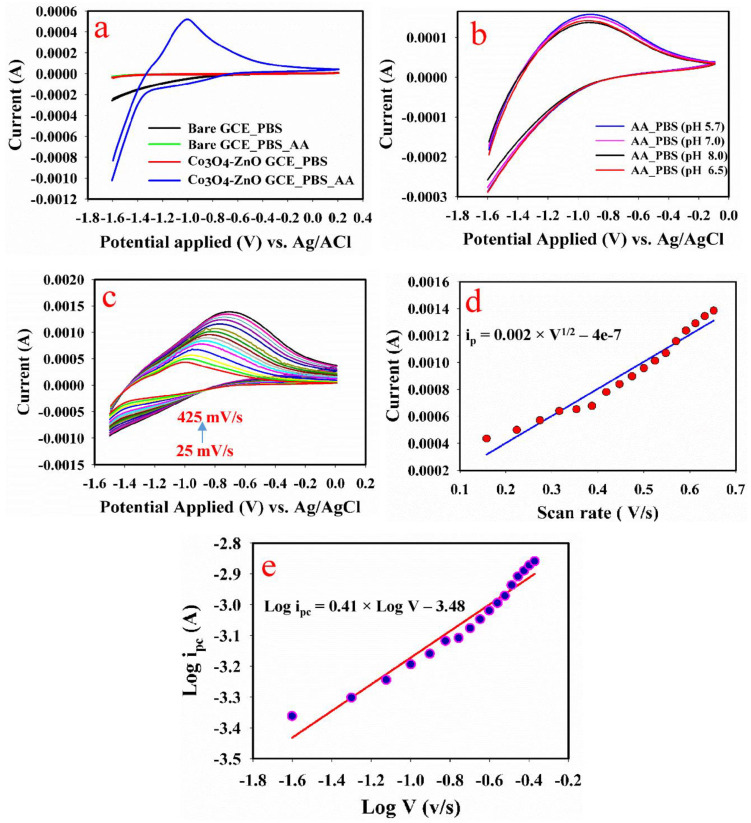
(**a**) Current response of Co_3_O_4_−ZnO nanorod modified GCE and bare GCE in PBS with/without presence of AA. (**b**) Current response of Co_3_O_4_−ZnO nanorod in PBS/AA at different pHs. (**c**) Current response of Co_3_O_4_−ZnO nanorod in PBS/AA at different scan rates (25–425 mV/s). (**d**) Plot of square root of scan rate versus oxidation peak current. (**e**) Plot of logarithm of oxidation peak current versus logarithm of scan rate.

A control study was conducted to establish that the current response demonstrated by the Co_3_O_4_-ZnO nanorod modified GCE was due to the Co_3_O_4_-ZnO nanorod substrate (coated material) and not the carbon in the GCE. As compared with the bare GCE, the coated GCE displayed a sharp peak at −0.95 V, signifying the oxidation of aspartic acid at this potential (Figure 4a). The result indicates that the oxidation of aspartic acid was due to the coated substrate.

#### 3.8.2. pH Variation

In most electro-catalyzed reactions, not only is the coated substrate responsible for the catalytic behavior, but the pH also plays an important role. The result of the control experiment is presented in Figure 4b. For this study, phosphate-buffered solution (PBS) of different pH was used (5.7, 6.5, 7.0, and 8.0). The result reveals that the PBS of pH 5.7 had the highest oxidation current while the PBS of pH 8 had the lowest oxidation current.

#### 3.8.3. Scan Rate Effect

Different scan rates ranging from 25 mV/s to 425 mV/s were applied to determine whether the oxidation of aspartic acid on Co_3_O_4_-ZnO nanorod is diffusion-controlled or not. The obtained result is presented in Figure 4c. It could be found the oxidation current (i_p_) increased linearly with increasing scan rate. This suggests that the reaction is diffusion-controlled [39,40,41]. In addition, the plot of the logarithm of the oxidation current (log i_p_) against the logarithm of the scan rate (log v) had a slope of 0.41. As given in the literature [42], the slope of log i_p_ vs. log v in the range of 0.5 is associated with the diffusion control process, while the one in the range of 1.0 is an adsorption-controlled process. Therefore, for this analysis, the slope is close to 0.5, which indicates that the reaction is diffusion-controlled.

#### 3.8.4. Calibration Plot

The linear sweep voltammetric (LSV) method was utilized to evaluate the current response to varying concentrations of aspartic acid. LSV was selected owing to its high accuracy, as well as its sensitivity, especially to irreversible reactions [43,44].

**Figure 5:**
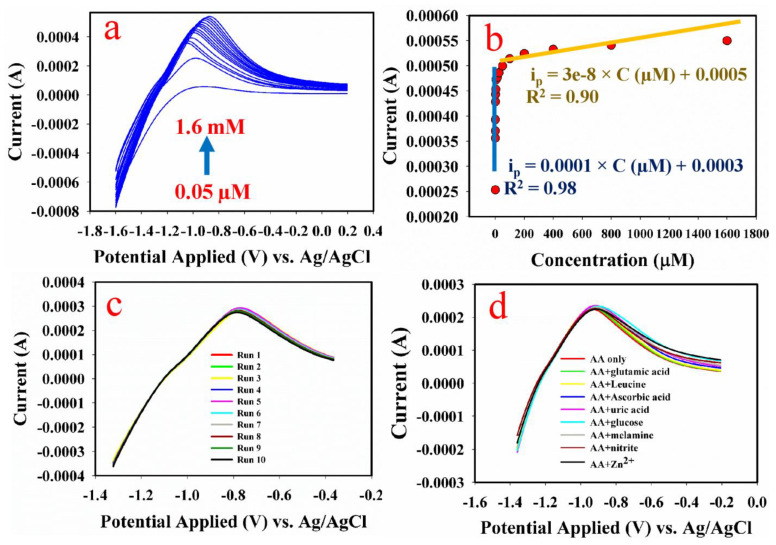
(**a**) Current response of Co_3_O_4_−ZnO nanorod modified GCE, increasing with AA concentration. (**b**) Plot of oxidation peak current against varying AA concentration. (**c**) Current response of Co_3_O_4_−ZnO nanorod in 0.05 µM AA taken 10 consecutive times. (**d**) Current response of Co_3_O_4_−ZnO nanorod in 0.05 µM AA with/without likely interfering species (glutamic acid, leucine, ascorbic acid, uric acid, glucose, melamine, nitrite and Zn^2+^).

The obtained linear sweep voltammogram is presented in Figure 5a. From the cyclic voltammetry, it can be seen that the oxidation peak current increased with the increase in aspartic concentration. For the calibration plot, two slopes were obtained, as revealed in Figure 5b. The two slopes obtained indicate that at higher concentrations, the electrocatalytic properties of the substrate (Co_3_O_4_-ZnO nanorod) diminished, which could be a result of the blockage of the active sites of the substrate. In this reaction [45], the Co_3_O_4_-ZnO nanorod, owing to its electrical conductivity and catalytic potential, electro-catalyzed the oxidation process, leading to an observed increment in the oxidation peak current. In this instance, it is proposed that Co (IV) and Zn (II) are reduced to zero-valent Co and Zn in the acidic medium (pH 5.7). The electrochemical redox reaction in the GCE substrate is proposed in the following reactions (Equations (2) and (3)).

Co_3_O_4_ + [Asp-A] + 8H^+^ = 3Co + 4H_2_O (2)

ZnO + [Asp-A] + 2H^+^ = Zn + H_2_O (3)

The calibration plot was used for investigating the analytical sensor performance of the fabricated sensor probe.

#### 3.8.5. Analytical Performance

The proposed sensor based on a Co_3_O_4_-ZnO nanorod was assessed using several parameters, such as the limit of detection (LOD), the limit of quantification (LOQ), sensitivity, etc.

LOD: The limit of detection was calculated as presented in Electronic Supplemental Section S2. The obtained value was 0.032 µM.

LOQ: The limit of quantification was calculated as described in Electronic Supplemental Section S2. The obtained value was 0.11 µM.

Sensitivity: The sensitivity of the proposed sensor was evaluated as described in Electronic Supplemental Section S2. The obtained value was 0.0014 µA µM^−1^ cm^−2^.

#### 3.8.6. Sensor Stability

The stability of the proposed fabricated sensor probe was evaluated in terms of current response repeatability and the interference effect.

For the repeatability study, ten consecutive readings were recorded as displayed in Figure 5c. The current responses were very repeatable with a relative standard deviation of 2.1%. Moreover, the effects of likely interfering species such as electroactive compounds, selected types of amino acid and metal ions were investigated (Figure 5d). The relative standard deviation of the oxidation current obtained with and without interference was 2.4%. This value is less than 5% of the adjudged interference threshold RSD [25].

#### 3.8.7. Real Sample Analysis

Real sample analysis was conducted in this study using a beverage drink and mouse serum. The results obtained are presented in Table 1. The percent recovery of the spiked aspartic concentration ranged from 87.6% to 94.4% (as calculated in Electronic Supplemental Section S3). This result indicates that the proposed method is suitable for real sample analysis.

#### 3.8.8. Comparability with Other Reported Methods

As indicated earlier, several methods have been investigated for the determination or detection of aspartic acid. These are presented in Table 2. Most of the earlier methods reported were based on the fluorescence technique and generally showed high accuracy. The proposed method based on the Co_3_O_4_-ZnO nanorod compares very well with the reported studies [1,2,46,47,48,49,50,51,52,53]. In addition, the proposed method displays higher stability and suitability for real sample analysis.

## 4. Conclusions

In this study, a composite Co_3_O_4_-ZnO nanorod was prepared by the facile wet chemical approach in alkaline phase at low temperature. The synthesized nano-metal oxide composite was well-characterized by different techniques, which confirms its successful synthesis. The Co_3_O_4_-ZnO nanorod was used for the electrochemical detection of aspartic acid through the electrocatalytic oxidation reaction on the surface of the modified GCE. The developed sensor had a low limit of detection, as well as high sensitivity and selectivity towards aspartic acid. Finally, the developed sensor was used for the detection of aspartic acid in real samples (beverage drink and mouse serum) with satisfactory performance. Therefore, this study presents a promising technique for the potential detection of aspartic acid for healthcare management.

## Figures and Tables

**Table 1:** Results of real sample analysis with a Co_3_O_4_-ZnO nanorod by the electrochemical method.

Sample Type	N/A	Spiked (µM)	Found (µM)	RSD (%)	Bias	Recovery (%)
Beverage drink	3	0 µM	N.D	N.D	-	N.D
	3	50 µM	47.2 ± 1.48	3.14	−2.8	94.4
Mouse serum	3	0 µM	N.D	N.D	N.D	N.D
	3	50 µM	43.8 ± 1.73	3.95	−6.2	87.6

N.D: not detected; N/A: number of analyses.

**Table 2:** Earlier-reported methods for the detection of aspartic acid and their analytical performance.

Sensing Matrix	Method	LOD (µM)	^#^ LR (µM)	Ref.
Dansyl derivative/SDS	Fluorescence	0.60	0.04–0.8mM	[46]
Lanthanide complex	Fluorescence	0.46	0.1–1000	[47]
Bifunctional materials	Fluorescence	0.26	0.1–133	[48]
N-Doped carbon	Fluorescence	0.09	0.5–50	[1]
Glucosamine-cyanine probe	Fluorescence	-	0.1–10	[49]
2-(2-pyridyl) benzimidazole	Fluorescence	10	10–300	[50]
Flurescent Cu (II) ion	Fluorescence	0.084	1–10	[51]
Ag_2_O/ZnO	Electrochemical	3.5	15–105	[52]
Polymer/MWCNTs	Electrochemical	4.08 ng/mL	3.89–66.23	[2]
Bolvin-serum/TiO_2_/GCE	Electrochemical	0.009	-	[53]
Co_3_O_4_-ZnO/GCE	Electrochemical	0.03	0.05–50	Current study

^#^ LR: linear range.

## Data Availability

Data will be available upon reasonable request.

## Supplementary Materials

The following supporting information can be downloaded at: https://www.mdpi.com/article/10.3390/bios13010088/s1, Electronic Supplemental Section: S1, S2 and S3.
