# Supplementary material for: Sensitive and Rapid Detection of Aspartic Acid with Co3O4-ZnO Nanorods Using Differential Pulse Voltammetry
Source: Biosensors (Basel). 2023 Jan 5;13(1):88. doi: 10.3390/bios13010088 (PMC9855673; doi:10.3390/bios13010088)
Supplement: Supplementary file 1 [file biosensors-13-00088-s001.zip › biosensors-2067249-supplementary.pdf]

biosensors

MDPI

Supporting Information

Sensitive and Rapid Detection of Aspartic Acid with Co_3O_4-ZnO Nanorods Using Differential Pulse Voltammetry

Sulaiman Y. Alfaifi [1], Waheed Abiodun Adeosun [1,2], Abdullah M. Asiri [1,2] and Mohammed M. Rahman [1,2,*]

[1] Chemistry Department, Faculty of Science, King Abdulaziz University, P.O. Box 80203, Jeddah 21589, Saudi Arabia
[2] Center of Excellence for Advanced Materials Research (CEAMR), King Abdulaziz University, P.O. Box 80203, Jeddah 21589, Saudi Arabia
* Correspondence: mmrahman@kau.edu.sa

Electronic Supplemental Section

S1 (a)

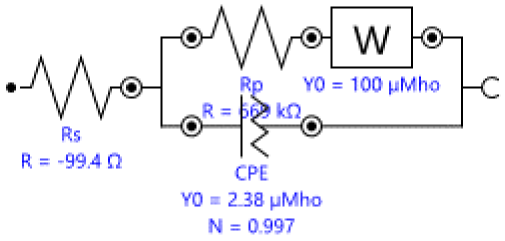

Element	Parameter	Value
Rs	R	−99.356
Rct	R	6.6851×10^5
CPE	Y0	2.3807×10^{-6}
	N	0.99701
W1	Y0	0.0001

(b)

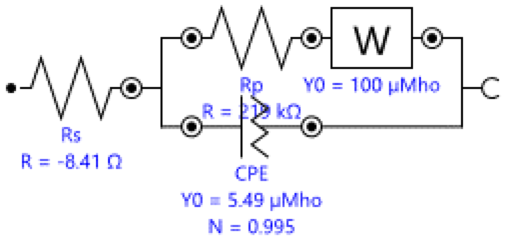

Element	Parameter	Value
Rs	R	−8.4112
Rp	R	2.1882×10^5
CPE	Y0	5.4865×10^{-6}
	N	0.99533
W1	Y0	0.0001

S2: Analytical Performance Parameters

Limit of Detection (LOD)

This was calculated using the equation;

$$\text{LOD} = \frac{3 \text{ X SD}}{\text{Slope of the calibration plot}}$$

where SD is the standard deviation of the blank response.
From our results, LOD = $3 \times 1.05 \times 10^{-6}$/ (0.0001) = 0.032 μM

Limit of Quantification (LOQ)

This was determined using the equation.

$$\text{LOQ} = \frac{10 \text{ x SD}}{\text{Slope of the calibration plot}}$$

LOQ = $10 \times 1.05 \times 10^{-6}$/ (0.0001) = 0.11 μM

Sensitivity

This was calculated using the formula:

$$\textbf{Sensitivity} = \frac{\textbf{Slope of the calibration plot}}{\textbf{GCE surface area}}$$

Sensitivity = 0.0001 $\mu A \mu M^{-1}$ / 0.07 cm^2 = 0.0014 $\mu A \mu M^{-1} cm^{-2}$

S3: Real Sample Analysis

Method used: Standard addition.

$$\text{Recovery (\%)} = \frac{\text{Ci}-\text{Co}}{\text{Cs}}$$

Where Ci is the total concentration of analyte, Co is concentration of analyte (before spike), Cs is the concentration of the spiked 2, 4-dnphz.
